# The Relationship between the JobMatchTalent Test and the NEO PI-R: Construct Validation of an Instrument Designed for Recruitment of Personnel

**DOI:** 10.1371/journal.pone.0090309

**Published:** 2014-03-04

**Authors:** Danilo Garcia, Ali Al Nima, Catrin Rappe, Max Rapp Ricciardi, Trevor Archer

**Affiliations:** 1 Network for Empowerment and Well-Being, University of Gothenburg, Gothenburg, Sweden; 2 Center for Ethics, Law and Mental Health (CELAM), University of Gothenburg, Gothenburg, Sweden; 3 Department of Psychology, University of Gothenburg, Gothenburg, Sweden; University of Gothenburg, Sweden

## Abstract

**Background:**

Personality measures in recruitment situations need to (1) cover the Big-Five model of personality and (2) focus on interpersonal requirements of jobs. We investigated the relationship between the JobMatchTalent test and the NEO Personality Inventory-Revised (NEO PI-R). The JobMatchTalent consists of three areas (i.e., Stability Patterns, Action Patterns, and Relation Patterns) divided in 10 main scales providing a deeper picture of the employee (e.g., Work Structure, Tolerance).

**Method:**

The participants (*N* = 390) were recruited from the professional network LinkedIn and completed online versions of both instruments. We used correlation analysis to investigate the construct validity of the JobMatchTalent test by identifying significant correlation coefficients no lower than ±.30 (i.e., convergent validity) and those with nonsignificant correlations (i.e., discriminant validity). Regression analyses were used to investigate the variance of the NEO PI-R dimensions that was explained by the JobMatchTalent test.

**Results:**

Four of the NEO PI-R dimensions showed considerable overlap with the following JobMatchTalent main scales: (1) Work structure and Decision Characteristics, which both are measures of thoughtfulness, planning, and order (i.e., Conscientiousness); (2) Inner drive, Activity, Drive, Acting, and Communication, which represent different aspects of being outgoing and extrovert (i.e., Extraversion); (3) Tolerance and Social interest, which measure a person's interest and ability to create social relations (i.e., Agreeableness); and (4) Stress Index, a measure of emotional stability (i.e., the opposite of Neuroticism). All 5 NEO PI-R dimensions overlapped with the JobMatchTalent sub-scales.

**Conclusions:**

The study suggests that 4 of the NEO PI-R dimensions are logically categorized along the JobMatchTalent main scales: (1) Order and Thoughtfulness, (2) Energy and Extraversion, (3) Social Adaptation and Interest, and (4) Emotion Control. Hence, it suggests substantial overlap between the instruments, but also that the two instruments cannot be considered as equivalent to assess individual differences in recruitment situations.

## Introduction

Personality was early defined as “factors”, such as genetically influenced dispositions and interpersonal strategies, within the individual explaining her/his behavior [1]. Others like Cloninger [2] have developed definitions of personality taking into account the unique ways in which individuals express themselves and adapt to the environment. Regardless of definition, personality measurement has been proposed as an important approach in the recruitment of personnel [3], [4]. Several measures have been developed for use in recruitment situations. A major suggestion in this context is that a personality test needs to at least contain reliable and valid scales for the standard five dimensions in the Big-Five model of personality (e.g., [3]).

The Big-Five model of personality comprises factors defined by inter-correlated traits or facets [5]. The model is often measured using the NEO Personality Inventory-Revised (NEO PI-R) which operationalizes the five dimensions: Openness (Facets: fantasy, aesthetics, feelings, actions, ideas, and values), Conscientiousness (Facets: competence, order, dutifulness, achievement striving, self-discipline, and deliberation), Extraversion (Facets: warmth, gregariousness, Assertiveness, activity, excitement-seeking, and positive emotions), Agreeableness (Facets: trust, straightforwardness, altruism, compliance, modesty, and tender-mindedness), and Neuroticism (Facets: anxiety, angry and hostility, depression, self-consciousness, impulsiveness, and vulnerability).

As suggested by others [3], some of the five dimensions in the Big-Five model might be irrelevant in the prediction of relevant work-related variables, such as performance in a particular job. For validity studies, however, it is important to use an inventory covering the five dimensions in order to determine the relevant and irrelevant dimensions [3]. For instance, Conscientiousness is one of the best predictors of job performance in the USA and Europe [6], [7]. In other words, an individual defined as effective, organized, ambitious, hardworking, and thoughtful is the most productive worker. Moreover, Extraversion (i.e., being sociable, talkative, confident, energetic, adventurous, and enthusiastic) together with Conscientiousness predicts job performance in various occupations [8]. In recruitment settings, however, managers and human resources experts describe characteristics such as stress tolerance, persistence, self-control, ability to cooperate and take initiative, and willingness to listen as important characteristics for job performance [3]. In order to facilitate the use of personality measures in recruitment situations instruments need to, besides covering the Big-Five dimensions, to focus on the personality and interpersonal requirements of different jobs [3].

The aim of the present study was to investigate the relationship between the NEO PI-R and the JobMatchTalent test, an instrument developed to measure individuals' work-related characteristics and then match these against specific demands related to specific occupations [9]. More than 25,000 people have done the test since the early 2000s and the test has been continuously developed to best meet the needs of managers and recruiters in their daily work with people. Nevertheless, to the best of our knowledge there are no published studies investigating the validity of the JobMatchTalent test. Thus, we briefly review the rational procedure in the development of the JobMatchTalent instrument and also describe its scales and sub-scales.

### JobMatchTalent

Klaus Olsen developed the JobMatchTalent test in collaboration with a group of recruitment consultants who wanted a test especially adapted to the needs that arose in their work. Olsen used panels of experts (e.g., managers, recruiters) and workers to get input in the early development stages of the JobMatchTalent test (for a description of this procedure, called “relevance check”, see [10]). This approach is important when tests are constructed to avoid questions that are only theoretically based (see [11], who criticize solely theoretical based instruments). In order to find suitable work-related personality traits, Olsen used various well-known personality instruments such as: the Myers- Briggs Type Indicator (a test that measures the different psychological types based on Carl Gustav Jung's theories [12]), the Sixteen personality factor Questionnaire (an instrument that measures 16 personality traits derived from factor analysis [13]), the Minnesota Multiphasic personality Inventory (an instrument that identifies the structure of personality and psychopathology [14]), and the NEO PI-R [15].

The instrument consists of three areas/domains that provide a broad picture of the individual's characteristics. These areas are called Stability Patterns, Action Patterns, and Relationship Patterns. These areas are divided into 10 main scales, which in turn comprise 30 sub-scales measuring work related traits that provide a deeper picture of the worker. The 10 main scales are: Work Structure, Inner Drive, Stress Index, Decisions Characteristics, Activity, Drive, Acting, Tolerance, Social Interest, Communication. See Table 1 for descriptions of each scale and its sub-scales.

**Table 1:** Description of the 10 main scales in the JobMatchTalent test and the name of the sub-scales for each main scale.

Domain	Main Scale	Description
Stability Patterns	Work Structure	Shows the degree of ability to prioritize and organize one's work. The sub-scales are: focus on planning, focus on details, and focus on order.
	Inner Drive	Measures the individual's ability to stay focused and motivated based on intrinsic motivation. The sub-scales are: self-motivation, optimism, and mood stability.
	Stress Index	It serves as an index of emotional stability at work despite outer influences such as stress, disturbance, and interpersonal provocations. The sub-scales are: self-control, resilience and concentration ability.
	Decisions Characteristics	Measures the individual's ability to make thoughtful and strong decisions and sticking with them. The sub-scales are: thoughtfulness, willpower, and persistence.
Action Patterns	Activity	Measures the level of energy put into work and the pace kept when performing the tasks. The sub-scales are: physical activity, mental energy, and need for speed.
	Drive	Measures the tendency to be driven by ambitions and degree of striving and aiming for success at work. The sub-scales are: winning instinct, vision, and development motivation.
	Acting	Measures an individual proneness to be proactive. The sub-scales are: sphere of influence, power of initiative, risk taking.
Relationship Patterns	Tolerance	An index of tolerance and trust in interpersonal relations. The sub-scales are concurring image, tolerant attitude, and trust in others.
	Social Interest	Measures the individual's interest in social interpersonal relations. The sub-scales are: displayed consideration, diplomacy and contact creating.
	Communication	Measures the general willingness and interest to express oneself to the external world. It is represented by the underlying degree of directness and strength in the communication, and the level of interest in actually communicating with others and the willingness to openly express one's opinion without restrain. The sub-scales are: focus in communication, communicativity, and openness.

### The present study

The purpose of the study was to identify convergent and discriminant correlations between the JobMatchTalent and NEO PI-R instruments by:

- Identifying significant correlations coefficients no lower than ±.30 (i.e., convergent validity) as recommended by Cohen [16] as a minimum effect size presenting a “practically” significant effect for social science data (see also Ferguson [17], who recommended an *r* = .2 as minimum effect size).
- Examining the content of the definitions of the scales and dimensions showing a correlation coefficient of ±.30 and above (i.e., convergent validity).
- Inspecting the content of the definitions of the scales and dimensions showing non-significant correlations (i.e., discriminant validity).

Although this study was exploratory in nature, we expected to find significant positive correlations between the Big-Five Conscientiousness dimension and the Work Structure and Decision Characteristics main scales of the JobMatchTalent test. That is, individuals who are effective, organized, ambitious, hardworking, and thoughtful (i.e., high in the Conscientiousness Big-Five dimension) should also report a high degree of ability to prioritize and organized their work by planning, paying attention to details, and order (i.e., Work Structure); as well as report high ability to make strong and thoughtful decisions (i.e., Decision Characteristics). Furthermore, we also expected the Extraversion dimension of the Big-Five model to be significantly positively correlated to the following JobMatchTalent main scales: Inner Drive, Activity, Acting, and Communication. Specifically, individuals who are sociable, talkative, confident, energetic, adventurous, and enthusiastic (i.e., high in the Extraversion Big-Five dimension) were expected to report optimistic emotions and intrinsic motivation (Inner Drive), high levels of energy and high pace at work (Activity), being able to take initiative and the willingness to cope with risk (Acting), and high ability and interests to communicate with others by openly expressing their opinion without restrain (Communication).

As described in the introduction, Conscientiousness and Extraversion are the two best predictors of job performance in various occupations [6]–[8]. Nevertheless, another expected significant positive correlation was that between the Agreeableness dimension of the NEO PI-R (i.e., the proneness to be trusting, sincere, generous, tolerant, modest, and empathic) to the Tolerance (i.e., the ability to have a uncritical attitude towards others and trusting others) and the Social Interest (i.e., the ability to display consideration to others, being diplomatic, and create and keep close contact with others) JobMatchTalent main scales. Finally, a negative correlation was expected between the NEO PI-R Neuroticism dimension (i.e., the proneness to experience negative emotions and being emotionally unstable) and the JobMatchTalent main scales called Stress Index (i.e., the aptitude to cope with stress and emotional stability at work despite outer influences) and Inner Drive (i.e., the proneness to optimistic emotions and intrinsic motivation).

## Method

### Ethics statement

This research protocol was approved by the Ethics Committee of the University of Gothenburg and written informed consent was obtained from all the study participants.

### Participants and procedure

Participants were recruited from LinkedIn (http://www.linkedin.com) which is a website mainly used for professional networking. The study was promoted through advertising, between the 16th of July of 2013 and the 31th of August of 2013, directed towards all Swedish users on LinkedIn. The advertising explained that LinkedIn users were invited to participate in a scientific study in collaboration with the University of Gothenburg, Sweden. The invitation also informed potential participants that they would receive their results from the JobMatchTalent test for free.

Participants registered by clicking on the invitation in LinkedIn and then through an online registration website in which they were informed about the study (e.g., assuring confidentiality, that their participation was voluntary, etcetera). Firstly, participants were asked to respond to questions regarding e-mail address, age, gender, and education. Secondly, participants were presented with the online version of the NEO PI-R. After completion of the NEO PI-R, participants received an e-mail containing login information and a link for the online version of the JobMatchTalent test. When the participants had completed the test they could click on a link and receive the results from the JobMatchTalent test.

A total of 566 individuals answered to the demographic questions and the NEO PI-R. Of these participants, 390 (125 males and 265 females) completed the JobMatchTalent test as well. Data from these 390 individuals (age mean: 39.54, *sd* 12.89), corresponding to 69% of those who originally agreed to participate, are used in the analyses presented here. For these 390 individuals education was as follows: 26 Grade School, 34 Basic Education for adults, 31 Vocational Education, 285 College/University, and 14 Higher Education.

### Measures

#### NEO PI-R [15]

The Swedish version of NEO PI-R was used to assess personality according to the Big-Five model of personality (for some studies using the Swedish version see [18], [19]). The NEO PI-R comprises 240 items, with 8 items to measure each personality facet using a 5-point Likert scale (1 =  strongly disagree, 5 =  strongly agree). Examples of the items are: “I have a very active imaginative skill” (Openness), “I am known for carefulness and common sense (Conscientiousness), “I work and play in an unhurried style” (Extraversion), “I try to be polite to anyone I meet” (Agreeableness), and “I get scared easily” (Neuroticism). The mean score of each facet was summarized to create each of the 5 dimensions. In the present study, the NEO PI-R showed Cronbach's alphas between .86 and .93 for the Big Five dimensions.

#### JobMatchTalent test [9]

The Swedish version of the JobMatchTalent test was used to assess work related personality characteristics. The JobMatchTalent test comprises 200 items, using a 5-points scale (1 =  *No/Disagree*, 5 =  *Yes/Agree*), organized in 10 main scales and 30 sub-scales. Examples of the items are: “Do you generally work according to predetermined plans?” (Work Structure), “I should probably be better at keeping myself going and staying alert” (Inner Drive), “Do you easily get temperamental, rather than try to keep calm at all times?” (Stress Index), “One of my key principles is to consider things carefully to ensure that all aspects are included” (Decision Characteristics), “Are you generally so engaged that you tend to carry on with various activities a bit too late in the evening?” (Activity), “Are you usually so resolute to accomplish a certain thing that you are willing to cross the line to reach the goal?” (Drive), “Currently I hold a leading position and/or initiating role within a project or a company” (Acting), “Are you usually critical about the things you observe or experience?” (Tolerance), “Are you so involved in your work and career that you only devote a minimum of your time to family and friends?” (Social Interest), “When meeting colleagues or friends, do you often talk about things that have recently happened” (Communication). The JobMatchTalent scores were standardized (*T-Scores*) as described in the technical manual of the instrument (Olsen, 2013). In the present study the Cronbach's alphas for the main scales were between .51 and .89. The Activity main scale and Decision Characteristics showed the lowest reliability coefficients (.51 respectively .54), while the rest showing from .69 and above.

### Statistical treatment

Before the main analyses, we conducted an Analysis of Variance using the Big-Five dimensions as the dependent variables in order to control for any important difference between the group of participants who only completed the NEO PI-R and the group of participants who completed both tests. No differences in personality dimensions were found between these two groups (*F*(5, 557) = .32, *p* = .902, *Wilks' Lambda*  = .997). Suggesting that those who completed both tests could be seen as representative for the original group and that the dropout was not dependent on their personality, at least in terms of the Big-Five dimensions.

The analyses were done in two steps. The first step was to conduct correlation analysis between JobMatchTalent's 10 main scales and NEO PI-R's 5 dimensions. The second step was the correlation of the JobMatchTalent's 30 sub-scales and NEO PI-R's 5 dimensions. The relationship between the instruments was analyzed using Pearson correlations in both steps. In order to investigate the variance of the NEO PI-R dimensions that could be explained by the JobMatchTalent's main we also conducted Multiple Regression Analyses (MRA) using all the JobMatchTalent main scales (and sub-scales in the second step of the analysis) as predictors and each NEO PI-R dimension as the dependent variable. All analyzes were conducted using SPSS (version 20).

## Results and Discussion

### Correlation analysis between JobMatchTalent main scales and the NEO PI-R dimensions

All 10 JobMatchTalent main scales had significant correlations above ±.30 with 4 of the NEO PI-R dimensions: Conscientiousness, Extraversion, Agreeableness, and Neuroticism (see Table 2). Nevertheless, each of the Big-Five dimensions also showed non-significant associations to specific JobMatchTalent main scales (e.g., Extraversion was not significantly related to Work Structure and Stress Index). Thus, showing construct validity (convergent and discriminant) between these NEO PI-R dimensions and specific JobMatchTalent main scales. As expected, Conscientiousness was positively correlated to Work Structure (*r* = .50, *p*<.001) and Decision Characteristics (*r* = .49, *p*<.001). Using an MRA, we found that the JobMatchTalent's main scales explained a significant proportion of variance in Conscientiousness scores (Adjusted *R^2^* = .39, *F*(10, 379) = 25.94, *p*<.001). The significant predictors were: Work Structure (β = .44, *t*(379) = 8.50, *p*<.001), Decision Characteristics (β = .11, *t*(379) = 1.48, *p*<.01), and Acting (β = .16, *t*(379) = 2.75, *p*<.01). Suggesting that, Conscientiousness might also be an indicator of the attributes measured by the Acting scale. The Acting main scale of JobMatchTalent measures an individual proneness to be proactive.

**Table 2:** Correlations between the JobMatchTalent main scales and the NEO PI-R dimensions.

		JOBMATCH TALENT									
		Stability Patterns				Action Patterns			Relationship Patterns		
		Work structure	Inner drive	Stress index	Decision Characteristics	Activity	Drive	Acting	Tolerance	Social interest	Communication
**NEO PI-R**	Openness	−.07▪	.21**	−,02▪	−.09▪	.22**	.21**	.21**	.12*	.17**	.23**
	Conscientiousness	.50**○	.32**○	.28**	.49**○	.12*	.25**	.25**	.01▪	−.09▪	.10▪
	Extraversion	−.10▪	.51**○	.16**	.08▪	.60**○	.37**○	.48**○	.18**	.18**	.54**○
	Agreeableness	.08▪	.04▪	.14**	−.22**	−.14**	−.33**	−.20**	.56**○	.49**○	−.29**
	Neuroticism	−.09▪	−.57**○	−.62**○	−.24**	−.02▪	−.06▪	−.33**○	−.32**○	.01▪	−.03▪

Note: *p<.05, **p<.01.

○ Convergent correlations (*r* = ±.30),

▪ Discriminant correlations (non-significant).

Also as expected, Extraversion was significantly positively related to the following JobMatchTalent main scales: Inner Drive (*r* = .51, *p*<.001), Activity (*r* = .60, *p*<.001), Drive (*r* = .37, *p*<.001), Acting (*r* = .49, *p*<.01), and Communication(*r* = .54, *p*<.001). Using an MRA, we found that the JobMatchTalent's main scales explained a significant proportion of variance in Extraversion scores (Adjusted *R^2^* = 55, *F*(10, 379) = 48.64, *p*<.001). The significant predictors were: Inner Drive (β = .18, *t*(379) = 2.92, *p*<.01), Activity (β = .33, *t*(379) = 6.53, *p*<.001), Social Interest (β = .16, *t*(379) = 3.42, *p*<.01) and Acting (β = .40, *t*(379) = 7.81, *p*<.001). Nevertheless, also Work Structure (β = .11, *t*(379) = 2.56, *p*<.05) and Stress Index (β = .18, *t*(379) = 2.92, *p*<.01) predicted Extraversion, while Decision Characteristics counter predicted this NEO PI-R dimension (β = −.15, *t*(379) = −3.00, *p*<.01). The other end of this scale, Introversion, is positively associated to introspection and intelligence [20]. In the contrary, Extraversion is positively associated with risk- taking behaviors [21] and overconfidence [22], which might lead to poor decisions; thus, perhaps explaining the negative association between Decision Characteristics and Extraversion.

Agreeableness was significantly positively related to both the Tolerance (*r* = .56, *p*<.001) and the Social Interest (*r* = .49, *p*<.001) JobMatchTalent main scales. The MRA results indicated that the JobMatchTalent's main scales explained a significant proportion of variance in Agreeableness scores (Adjusted *R^2^* = .39, *F*(10, 379) = 25.85, *p*<.001). The significant predictors were: Tolerance (β = .45, *t*(379) = 6.57, *p*<.001) and Social Interest (β = .17, *t*(379) = 3.02, *p*<.01). Also Work Structure (β = .13, *t*(379) = 2.50, *p*<.05) predicted Agreeableness. In other words, this suggests that agreeable people might also be structured at work by keeping order and details in focus.

Finally, Neuroticism was significantly negatively correlated to the Inner Drive (*r* = −.57, *p*<.001) and the Stress Index (*r* = −.62, *p*<.001) JobMatchTalent main scales. The MRA results indicated that the JobMatchTalent's main scales explained a significant proportion of variance in Neuroticism scores (Adjusted *R^2^* = .48, *F*(10, 379) = 37.49, *p*<.001). The significant counter predictors were: Inner Drive (β = −.34, *t*(379) = −5.53, *p*<.001), Stress Index (β = −.37, *t*(379) = −6.49, *p*<.001), and Acting (β = −.18, *t*(379) = −3.42, *p*<.001). Nevertheless, Drive (β = .21, *t*(379) = 3.88, *p*<.001) and Social Interest (β = .11, *t*(379) = 2.23, *p*<.05) also predicted Neuroticism. While the Drive JobMatchTalent main scale measures the tendency to be driven by ambitions and degree of striving and aiming for success at work, the Social Interest main scale measures the general willingness and interest to express oneself to the external world. That both Drive and Social Tolerance predict a NEO PI-R dimension that identifies individuals who are prone to psychological distress (i.e., Neuroticism) is counterintuitive. However, recent field and experimental studies analyzing how well neurotics and extroverts succeed at work places organized in teams suggest that “Extraversion is associated with status losses and disappointing expectations for contributions to group tasks and Neuroticism is associated with status gains due to surpassing expectations for group-task” ([23], p. 387). Bendersky and Shah [23] suggested that workers high in Neuroticism are motivated to work hard on behalf of their teams because they perceive the low expectations put on them by other team members. This suggestion is in line with the results presented here, high levels of Neuroticism are related to personal characteristics such as striving and aiming for success at work (i.e., Drive) and express oneself to co-workers (Social Interest) in order to exhale the low expectations from others.

These results show the convergent and discriminant validity of the main scales of the JobMatchTalent test in relation to the personality traits measured by the NEO PI-R. Specifically, the main scales of JobMatchTalent created by Olsen seem to conceptualize the different dimensions of the NEO PI-R, with the exception of Openness. In order to analyze this further, we conducted the same type of analysis using the NEO PI-R dimensions and the JobMatchTalent sub-scales.

### Correlation analysis between JobMatchTalent sub-scales and the NEO PI-R dimensions

In this step of the analysis, all NEO PI-R dimensions showed a correlation coefficient of ±.30 or above with the JobMatchTalent sub-scales. As in the main scale vs. dimension analysis above, some of the correlations were non-significant. For example, Neuroticism was not significantly related to any of the sub-scales under Activity (i.e., Physical activity, Mental energy, and Need for speed). Indeed, the Activity main scale of the JobMatchTalent test was designed to measure the level of energy put into work and the pace kept when performing the tasks [9], while Neuroticism measures the proneness to experience negative emotions and being emotionally unstable. For instance, Neuroticism was strongly negatively correlated to the Mood stability sub-scale under the Inner Drive JobMatchTalent main scale. In contrast to the results between the JobMatchTalent's main scales and the NEO PI-R's dimensions, Openness showed correlations above .30 with three of the JobMatchTalent's sub-scales: Optimism, Vision, and Development motivation. Suggesting that high scores in these JobMatchTalent sub-scales describe an individual who is imaginative, inventive, enthusiastic, curious, optimistic, and unconventional (i.e., high in Openness as measured by the NEO PI-R, [15]). Thus, suggesting that the JobMatchTalent test has construct validity in relation to the 5 personality traits measured by the NEO PI-R at the sub-scale level. See Table 3 for the details.

**Table 3:** Correlations between the JobMatchTalent sub-scales and the NEO PI-R domain Scales.

			NEO PI-R				
			Openness	Conscientiousness	Extraversion	Agreeableness	Neuroticism
**JOBMATCH**	WORK STRUCTURE	Focus on planning	−.14^**^	.33^**○^	−.26^**^	.04▪	.01▪
		Focus on details	.04▪	.25^**^	−.07▪	.11^*^	.02▪
		Focus on order	−.03▪	.49^**○^	.09▪	.05▪	−.20^**^
	INNER DRIVE	Self-motivation	.01▪	.45^**○^	.42^**○^	−.23^**^	−.35^**○^
		Optimism	.43^**○^	−.11^*^	.44^**○^	.07▪	−.06▪
		Mood stability	−.02▪	.34^**○^	.22^**^	.14^**^	−.69^**○^
	STRESS INDEX	Self-control	−.09▪	.21^**^	−.08▪	.24^**^	−.47^**○^
		Resilience	.04▪	.22^**^	.23^**^	.07▪	−.62^**○^
		Concentration ability	.07▪	.16^**^	.26^**^	−.08▪	−.16^**^
	DECISION CHARACTERISTICS	Thoughtfulness	−.22^**^	.34^**○^	−.34^**○^	.04▪	−.04▪
		Willpower	.10^*^	.18^**^	.38^**○^	−.40^**○^	−.06▪
		Persistence	−.01▪	.35^**○^	.16^**^	−.03▪	−.34^**○^
	ACTIVITY	Physical Activity	.15^**^	.11^*^	.45^**○^	.05▪	−.04▪
		Mental energy	.21^**^	.31^**○^	.63^**○^	−.13^**^	−.21^**^
		Need for speed	.16^**^	−.07▪	.40^**○^	−.25^**^	.19^**^
	DRIVE	Winning instinct	.08▪	.31^**^	.35^**○^	−.37^**○^	−.09▪
		Vision	.32^**○^	.05▪	.31^**○^	−.23^**^	.01▪
		Development motivation	.30^**○^	.10^*^	.12^*^	.16^**^	−.01▪
	ACTING	Sphere of influence	.17^**^	.23^**^	.38^**○^	−.07▪	−.34^**○^
		Power of initiative	.16^**^	.19^**^	.43^**○^	−.29^**^	−.08▪
		Risk taking	.27^**^	−.06▪	.38^**○^	−.26^**^	−.10▪
	TOLERANCE	Concurring image	−.01▪	−.11^*^	−.14^**^	.48^**○^	−.03▪
		Tolerant attitude	.14^**^	.01▪	.30^**○^	.33^**○^	−.32^**○^
		Trust in others	.17^**^	.15^**^	.27^**^	.53^**○^	−.43^**○^
	SOCIAL INTEREST	Displayed consideration	.13^*^	−.21^**^	.02▪	.38^**○^	.14^**^
		Diplomacy	−.03▪	−.06▪	−.10^*^	.43^**○^	−.06▪
		Contact creating	.36^**○^	.14^**^	.72^**○^	.02▪	−.17^**^
	COMMUNICATION	Force in communication	.17^**^	.14^**^	.49^**○^	−.34^**○^	−.06▪
		Communicativity	.20^**^	−.01▪	.43^**○^	−.18^**^	.10▪
		Openness	.36^**○^	.15^**^	.61^**○^	.03▪	−.33^**○^

Note: *p<.05, **p<.01.

○ Convergent correlations (*r* = ±.30),

▪ Discriminant correlations (non-significant).

The first regression analysis showed that the JobMatchTalent's sub-scales explained a significant proportion of variance in Openness scores (Adjusted *R^2^* = .27, *F*(21, 368) = 7.73, *p*<.001). The significant predictors were: Vision, Development motivation, and Contact creating. Indeed, in addition to the discussion above, an individual high in Openness is also described as talkative, open-hearted, affectionate, and talkative, that is, with the ability to create relations to others (i.e., Contact creating in the JobMatchTalent test). The following JobMatchTalent sub-scales counter predicted Openness: Winning instinct and Force in communication. People high in Openness are suggested to actively negotiate conflicts while recognizing the other's perspective, thus, facilitating communication [24]. In contrast, people low in Openness are described as realistic and non-affectionate. In other words, low Openness at the work places is expressed by direct and dominant communication rather than being receptive for other people's point of view (see Table 4).

**Table 4:** Results of the regression analysis: the JobMatchTalent sub-scales in the prediction of the NEO PI-R dimensions.

Predictor	*β*	*Outcome*	*t*	*Adj. R2*	*F*
Winning instinct	−.17	Openness	−2.2*	.27	7.73***
Vision	.28		4.49***		
Development motivation	.22		4.56***		
Contact creating	.16		2.01*		
Force in communication	−.22		−2.11*		
Thoughtfulness	.30	Conscientiousness	5.30***	.39	12.95***
Willpower	.17		2.29*		
Persistence	.15		3.22**		
Mental energy	.23		2.94**		
Winning instinct	.22		3.01**		
Power of initiative	.19		2.63**		
Risk taking	−.30		−5.02***		
Thoughtfulness	−.12	Extraversion	−2.71**	.63	32.06***
Physical activity	.10		2.51*		
Mental energy	.20		3.19**		
Development motivation	−.07		−2.18*		
Power of initiative	−.12		−2.07*		
Tolerant attitude	.11		2.57*		
Contact creating	.45		8.06***		
Openness	.16		3.09**		
Physical activity	.11	Agreeableness	2.31*	.52	20.73***
Winning instinct	−.13		−2.01*		
Development motivation	.15		3.84***		
Risk taking	−.21		−4.06***		
Trust in others	.45		9.78***		
Displayed consideration	.20		3.49**		
Willpower	−.23	Neuroticism	−3.00**	.38	12.34***
Persistence	−.13		−2.75**		
Need for speed	.18		2.61**		
Concurring image	−.31		−2.82**		
Tolerant attitude	−.14		−2.48*		
Trust in others	−.21		−4.06***		
Displayed consideration	.18		2.77**		
Diplomacy	.27		3.03**		
Communicativity	.30		3.62***		
Openness	−.17		−2.65*		

Note: *p<.05, **p<.01, ***p<.001.

The second regression analysis showed that the JobMatchTalent's sub-scales explained a significant proportion of variance in Conscientiousness scores (Adjusted *R^2^* = .39, *F*(21, 368) = 12.95, *p*<.001). The significant predictors were: Thoughtfulness, Willpower, Persistence, Mental energy, Winning instinct, and Power initiative. All of which describe an individual's degree of organization, persistence, control and motivation in goal directed behavior or Conscientiousness [15]. This Big-Five dimension is negatively associated with impulsive sensation-seeking in Zuckerman's model [25] and with Novelty Seeking in Cloninger's model [26], which might explain the negative relationship between the Risk taking JobMatchTalent sub-scale and Conscientiousness (see Table 4).

The third regression analysis showed that the JobMatchTalent's sub-scales explained a significant proportion of variance in Extraversion scores (Adjusted *R^2^* = .63, *F*(21, 368) = 32.06, *p*<.001). The significant predictors were: Physical activity, Mental energy, Tolerant attitude, Contact creating, and Openness. Extroverts are indeed describes as energetic, talkative, and sociable [15]. The following JobMatchTalent sub-scales counter predicted Extraversion: Thoughtfulness, Development motivation, and Power of initiative. Suggesting that Introversion, the opposite of Extraversion, is related to being thoughtful but also to strive after one's own development and the ability to take initiative. As earlier discussed, Introversion, is positively associated to introspection and intelligence [20]. In contrast to the results presented here, extroverts should be expected to display a higher level of proactivity (i.e., Power initiative). Although introvert leaders, in contrast to extrovert leaders, promote initiative taking within teams composed by highly proactive co-workers [27]; the results presented here regarding Power initiative need replication before making plausible conclusions.

The next regression analysis showed that the JobMatchTalent's sub-scales explained a significant proportion of variance in Agreeableness scores (Adjusted *R^2^* = .52, *F*(21, 368) = 20.73, *p*<.001). The significant predictors were: Physical activity, Development motivation, Trust in others, and Displayed consideration. The following JobMatchTalent sub-scales counter predicted Agreeableness: Winning instinct and Risk taking. Indeed, the propensity to take risks has been associated to low Agreeableness and Conscientiousness [28].

The final regression analysis showed that the JobMatchTalent's sub-scales explained a significant proportion of variance in Neuroticism scores (Adjusted *R^2^* = .38, *F*(21, 368) = 12.34, *p*<.001). The significant predictors were: Need for speed, Displayed consideration, Diplomacy, and Communicativity. Individuals high in Neuroticism are described as tensed, restless, and excitable [15]. This is indeed closely connected to the need for speed at work and the downside of it, becoming easily irritated when things go too slow [9]. In line with Bendersky and Shah's suggestions [23], neurotics being motivated to work hard on behalf of their teams, it is plausible to concur that neurotics try to keep high speed also due to their focus on meeting teammates expectations. As discussed under the main scales vs. dimension section, high levels of Neuroticism are related to the need of expressing oneself to co-workers in order to exhale the low expectations from others, perhaps through diplomacy and showing consideration for others. Nevertheless, these results need to be interpreted with caution. The following JobMatchTalent sub-scales counter predicted Neuroticism: Willpower, Persistence, Concurring image, Tolerant attitude, Trust in others, Openness. These JobMatchTalent sub-scales do correspond to the opposite of Neuroticism, namely, Emotional Stability—described as being confident, trustful, and gentile. For the detailed results of all regression analysis see Table 4.

In sum, the aim of the present study was to investigate the relationship between the JobMatchTalent test and the NEO PI-R in order to identify the convergent and discriminant validity of the JobMatchTalent test. Specifically, we identified all significant correlations coefficients no lower than ±0.3 as recommended by Cohen [16] as a criterion for convergent validity. The non-significant correlations were assumed to represent the discriminant validity of the JobMatchTalent test in relation to the Big-Five dimensions. Further correlation analysis between the JobMatchTalent sub-scales and the NEO PI-R facets can be found in Table S1. The results of the second step show that the constructs in the JobMatchTalent sub-scales are valid measures of all 5 NEO PI-R dimensions. Nevertheless, Openness was only found to overlap with different JobMatchTalent's sub-scales, not the main scales.

### Limitations and suggestions for future research

We used a convenience sample in the present study, which might limit the generalizability of the findings. The sample was collected from LinkedIn, which is a website mainly used for professional networking. As described elsewhere, during recruiting situations respondents “manipulate responses to personality items to make a positive impression” ([29], p. 551). Thus, it is important to replicate the results presented here in real recruiting situations. Moreover, although the Big-Five has been regarded as a good basis for the design of tests for use in recruitment situations [3], factor analysis suggest that it has its limitations when screening personnel—a sixth factor related to individual's prior knowledge about the job has been found to appear [30]–[32]. This might explain some of the results from the regression analysis presented here. Nevertheless, two of the main scales in the JobMatchTalent test showed low reliability coefficients; which might have affected some of the results.

It is also plausible to discuss the rationale of the regression analysis conducted. From a theoretical point of view, it seems to make more sense to consider the NEO PI-R traits as predictors and interpersonal job requirements as criteria. However, the JobMatchTalent test was constructed to measure work-related personality traits by using personality instruments such as the Myers- Briggs Type Indicator [12], the Sixteen personality factor Questionnaire [13], and the Minnesota Multiphasic personality Inventory [14]. Thus, as in other studies testing personality instruments against each other [26], we opted to use the instrument to be validated as the predictor. Consequentially, the construct validity of the JobMatchTalent test could be investigated using other personality instruments that were not involved in its construction. The Temperament and Character Inventory [33], for example, includes measures of personal goals and values that guides peoples' behavior in their life (i.e., Self-directedness, Cooperativeness, and Self-transcendence); which might be compelling personality measures to validate the JobMatchTalent test against. Finally, the validity of the JobMatchTalent test in a selection procedure is questionable given the high relationships between some of its scales and some of the NEO PI-R's dimensions. Hence the best test of the usefulness of JobMatchTalent test would be using it and the NEO PI-R to compete in the prediction of some work criteria, for example, productivity (self-rated, manager-rated, and objectively-rated), perception of the work climate, motivation, organizational commitment, etcetera.

### Final remarks

**Figure 1:**
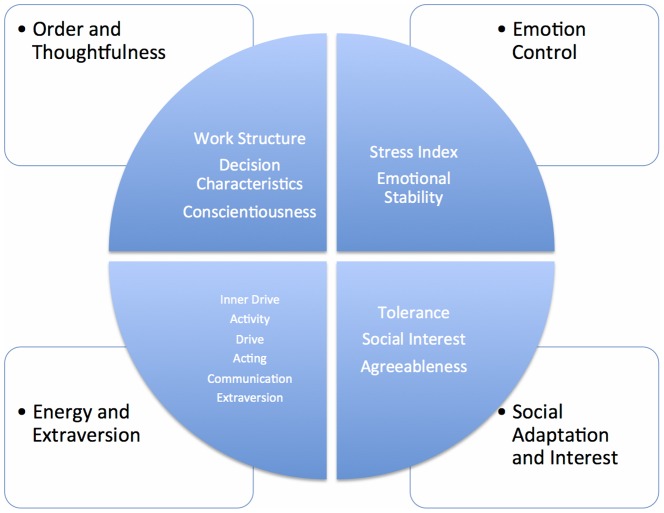
Illustration of the convergence validity results between the JobMatchTalent test and the NEO PI-R dimensions.

The results from the correlation analysis between JobMatchTalent main scales and NEO PI-R dimensions, suggest that 4 of the NEO PI-R dimensions are explained by (1) Work structure and Decision Characteristics, which both are measures of thoughtfulness, planning, order and details (i.e., Conscientiousness); (2) Inner drive, Activity, Drive, Acting, and Communication, which all represent different aspects of being outgoing and extrovert; (3) Tolerance and Social interest, which both measure a person's interest and ability to create social relations (i.e., Agreeableness); and (4) the Stress Index, which is a measure of emotional stability or the opposite of Neuroticism (see Figure 1). Conscientiousness is indeed associated with the desire to keep things organized and tidy and also with productivity and work ethic, and lower rates of absenteeism [34]–[36]. However, Extraversion, Agreeableness and Emotional Stability (i.e., the opposite end of Neuroticism) might also be important in jobs in which there is a significant amount of social interaction. Thus, the categories of characteristics and traits presented in Figure 1 might all be of importance for positive work-related outcomes [37].

## Conclusion

This study shows strong indication of significant convergent and discriminant validity between the JobMatchTalent test and the NEO PI-R. The study suggest that 4 of the 5 NEO PI-R dimensions can be discerned in a logical categorization along the JobMatchTalent characteristics: (1) Order and Thoughtfulness, (2) Energy and Extraversion, (3) Social Adaptation and Interest, and (4) Emotion Control. Moreover, at the subscale level, all 5 NEO PI-R dimensions overlapped with the JobMatchTalent sub-scales. Suggesting substantial overlap between the instruments, but also that the two instruments cannot be considered as equivalent to assess individual differences in recruitment situations.

*“I put all my genius into my*

*life; I put only my talent*

*into my works”*

*Oscar Wilde*

## Supporting Information

Table S1 Correlations between the JobMatchTalent sub-scales and the NEO PI-R facets.
(XLS)
